# Bis(3-methyl­pyridinium) tetra­(chlorido/bromido)cuprate(II)

**DOI:** 10.1107/S1600536811019076

**Published:** 2011-05-25

**Authors:** Young-Inn Kim, Hyun-Soo Lim, Sung Kwon Kang

**Affiliations:** a Department of Chemistry Education and Interdisciplinary Program of Advanced Information and Display Materials, Pusan National University, Pusan 609-735, Republic of Korea; b Department of Chemistry, Chungnam National University, Daejeon 305-764, Republic of Korea

## Abstract

The structure of the title salt, (C_6_H_8_N)_2_[CuCl_3.4_Br_0.6_], consists of two 3-methyl­pyridinium cations and a distorted tetra­hedral [CuCl_3.4_Br_0.6_]^2−^ dianion. Substitutional disorder with Br is exhibited for three of the Cl atoms of the anion, giving a mixed chloride/bromide cuprate(II) anion. In the crystal, inter­molecular N—H⋯Cl hydrogen bonds link two cations to one anion, forming a three-ion aggregate. These are connected into a supra­molecular chain along the *b* axis *via* π–π inter­actions between the pyridinium rings [centroid–centroid distance = 3.743 (3) Å].

## Related literature

For general background to the geometry of the tetra­halidocuprate(II) species, see: Solomon *et al.* (1992 ▶); Kim *et al.* (2001 ▶); Panja *et al.* (2005 ▶); Sengottvelan *et al.* (2009 ▶). For its magnetic properties, see: Lee *et al.* (2004 ▶); Turnbull *et al.* (2005 ▶); Shapiro *et al.* (2007 ▶). CuBr_4_^2−^ ions usually show less distortion from the ideal tetra­hedral geometry compared with CuCl_4_^2−^ ions, see: Edwards *et al.* (2011 ▶); AlDaman & Haddad (2011 ▶).
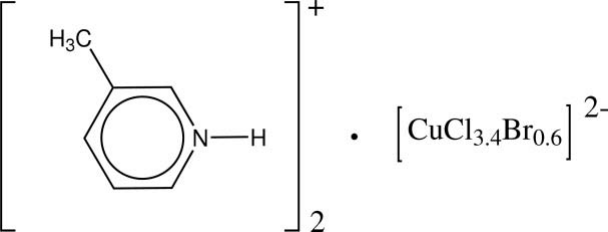


## Experimental

### 

#### Crystal data

(C_6_H_8_N)_2_[CuBr_0.60_Cl_3.40_]
*M* *_r_* = 420.28
Monoclinic, 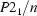

*a* = 9.0617 (18) Å
*b* = 13.259 (3) Å
*c* = 14.060 (3) Å
β = 102.47 (3)°
*V* = 1649.4 (6) Å^3^
*Z* = 4
Mo *K*α radiation
μ = 3.32 mm^−1^
*T* = 295 K
0.19 × 0.15 × 0.15 mm

#### Data collection

Bruker SMART CCD area-detector diffractometer
Absorption correction: multi-scan (*SADABS*; Bruker, 2002 ▶) *T*_min_ = 0.560, *T*_max_ = 0.610
17556 measured reflections
4094 independent reflections
2621 reflections with *I* > 2σ(*I*)
*R*_int_ = 0.039

#### Refinement

*R*[*F* ^2^ > 2σ(*F* ^2^)] = 0.035
*wR*(*F* ^2^) = 0.089
*S* = 1.03
4094 reflections
209 parameters
H atoms treated by a mixture of independent and constrained refinement
Δρ_max_ = 0.34 e Å^−3^
Δρ_min_ = −0.41 e Å^−3^

### 

Data collection: *SMART* (Bruker, 2002 ▶); cell refinement: *SAINT* (Bruker, 2002 ▶); data reduction: *SAINT*; program(s) used to solve structure: *SHELXS97* (Sheldrick, 2008 ▶); program(s) used to refine structure: *SHELXL97* (Sheldrick, 2008 ▶); molecular graphics: *ORTEP-3 for Windows* (Farrugia, 1997 ▶); software used to prepare material for publication: *WinGX* (Farrugia, 1999 ▶).

## Supplementary Material

Crystal structure: contains datablocks global, I. DOI: 10.1107/S1600536811019076/tk2745sup1.cif

Structure factors: contains datablocks I. DOI: 10.1107/S1600536811019076/tk2745Isup2.hkl

Additional supplementary materials:  crystallographic information; 3D view; checkCIF report

## Figures and Tables

**Table:** 

Cu1—Cl2	2.232 (8)
Cu1—Cl4	2.248 (10)
Cu1—Cl5	2.2604 (8)
Cu1—Cl3	2.273 (3)

**Table:** 

Cl2—Cu1—Cl4	97.5 (3)
Cl2—Cu1—Cl5	135.34 (14)
Cl4—Cu1—Cl5	99.0 (3)
Cl2—Cu1—Cl3	100.2 (2)
Cl4—Cu1—Cl3	135.8 (2)
Cl5—Cu1—Cl3	96.18 (8)

**Table 2:** Hydrogen-bond geometry (Å, °)

*D*—H⋯*A*	*D*—H	H⋯*A*	*D*⋯*A*	*D*—H⋯*A*
N1—H1⋯Cl5	0.76 (3)	2.50 (3)	3.158 (3)	145 (3)
N8—H8⋯Cl3	0.82 (3)	2.53 (3)	3.245 (4)	147 (3)
N8—H8⋯Cl5	0.82 (3)	2.72 (3)	3.332 (3)	133 (3)

**Fig. 1.**
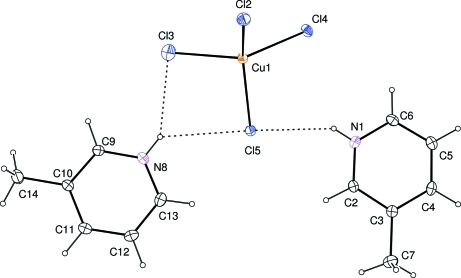
The molecular structure of the title compound, showing displacement ellipsoids drawn at the 30% probability level. The N—H···Cl hydrogen bonds are indicated with dashed lines.

**Fig. 2.**
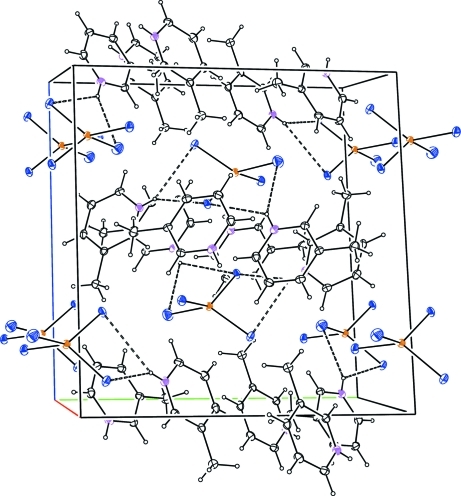
Crystal structure of viewed normal to (1 0 0), showing the N—H···Cl hydrogen bonds as dashed lines.

## supplementary crystallographic information

### Comment

The structural properties of tetrahalocuprate(II) compounds have attracted continued interest as model compounds for biological process (Solomon *et al.*, 1992; Kim *et al.*, 2001; Panja *et al.*, 2005) as well as magnetic functional materials (Lee *et al.*, 2004; Turnbull *et al.*, 2005; Shapiro *et al.*, 2007). In a previous paper, we (Sengottvelan *et al.*, 2009) reported the structure of bis(3-methylpyridinium)tetrachlorocuprate(II) and investigated packing interactions such as hydrogen bonding and π–π interactions. Because CuBr_4_^2-^ ions usually show less distortion from the ideal tetrahedral geometry compared with CuCl_4_^2-^ (Edwards *et al.*, 2011; AlDaman & Haddad, 2011), the analogous chemistry with CuBr_4_^2-^ was investigated. Herein, we disclose the the crystal structure of the bis(3-methylpyridinium) salt of a mixed-tetrachlorido/bromido cuprate(II) ion, [CuCl_3.4_Br_0.6_]^2-^.

The structure of the title salt, [C_6_H_8_N]_2_[CuCl_3.4_Br_0.6_], consists of two 3-methylpyridinium cations and one distorted tetrahedral [CuCl_3.4_Br_0.6_] anion. There are substitutional disorder for three of the Cl atoms anion to make a mixed-halo cuprate (II) anion. The [CuCl_3.4_Br_0.6_]^2-^ anion is has approximately D_2d_ symmetry, with the distortion from the ideal tetrahedral partly arising as a result of three different hydrogen bonding interactions with two 3-methylpyridinium cations (Fig. 1). The range of Cl—Cu—Cl angles is 96.18 (8) – 135.8 (2) ° (Table 1). There are weak aromatic π-π interactions between pyridinium rings of the discrete tri-ion aggregates [centroid-centroid distance = 3.743 (3) Å], and these lead to a supramolecular chain along the *b* axis.

### Experimental

Copper(II) chloride (1.36 g, 8 mmol) dissolved in ethanol, was added drop wise to a stirred ethanolic solution containing 3-methylpyridine (0.744 g, 8 mmol) and concentrated HBr (0.5 ml, 4.4 mmol). The mixture was refluxed for approximately 4 h at 333 K. The resulting solution was filtered and allowed to stand at room temperature. The crystals were obtained after 2–3 days.

### Refinement

The H1 and H8 atoms were located in a difference map and refined freely. Other H atoms are positioned geometrically and refined using a riding model, with C—H = 0.93 – 096 Å, and with *U*_iso_(H) = 1.2*U*_eq_(C) for aromatic and 1.5*U*_eq_(C) for methyl H atoms. Atoms Cl2, Cl3, and Cl4 are substitutionally disordered with Br atoms. The final occupancy factors for the Cl and Br atoms were fixed at 0.80 and 0.20, respectively.

### Crystal data

**Table:**

(C_6_H_8_N)_2_[CuBr_0.60_Cl_3.40_]	*F*(000) = 839.2
*M**_r_* = 420.28	*D*_x_ = 1.693 Mg m^−^^3^
Monoclinic, *P*2_1_/*n*	Mo *K*α radiation, λ = 0.71073 Å
Hall symbol: -P 2yn	Cell parameters from 3355 reflections
*a* = 9.0617 (18) Å	θ = 2.8–23.7°
*b* = 13.259 (3) Å	µ = 3.32 mm^−^^1^
*c* = 14.060 (3) Å	*T* = 295 K
β = 102.47 (3)°	Block, brown
*V* = 1649.4 (6) Å^3^	0.19 × 0.15 × 0.15 mm
*Z* = 4	

### Data collection

**Table:**

Bruker SMART CCD area-detector diffractometer	2621 reflections with *I* > 2σ(*I*)
graphite	*R*_int_ = 0.039
φ and ω scans	θ_max_ = 28.3°, θ_min_ = 2.1°
Absorption correction: multi-scan (*SADABS*; Bruker, 2002)	*h* = −10→12
*T*_min_ = 0.560, *T*_max_ = 0.610	*k* = −17→17
17556 measured reflections	*l* = −18→18
4094 independent reflections	

### Refinement

**Table:**

Refinement on *F*^2^	Primary atom site location: structure-invariant direct methods
Least-squares matrix: full	Secondary atom site location: difference Fourier map
*R*[*F*^2^ > 2σ(*F*^2^)] = 0.035	Hydrogen site location: inferred from neighbouring sites
*wR*(*F*^2^) = 0.089	H atoms treated by a mixture of independent and constrained refinement
*S* = 1.03	*w* = 1/[σ^2^(*F*_o_^2^) + (0.0389*P*)^2^ + 0.0822*P*] where *P* = (*F*_o_^2^ + 2*F*_c_^2^)/3
4094 reflections	(Δ/σ)_max_ = 0.001
209 parameters	Δρ_max_ = 0.34 e Å^−^^3^
0 restraints	Δρ_min_ = −0.41 e Å^−^^3^

### Special details

**Table:**

Geometry. All s.u.'s (except the s.u. in the dihedral angle between two l.s. planes) are estimated using the full covariance matrix. The cell s.u.'s are taken into account individually in the estimation of s.u.'s in distances, angles and torsion angles; correlations between s.u.'s in cell parameters are only used when they are defined by crystal symmetry. An approximate (isotropic) treatment of cell s.u.'s is used for estimating s.u.'s involving l.s. planes.
Refinement. Refinement of *F*^2^ against ALL reflections. The weighted *R*-factor *wR* and goodness of fit *S* are based on *F*^2^, conventional *R*-factors *R* are based on *F*, with *F* set to zero for negative *F*^2^. The threshold expression of *F*^2^ > 2σ(*F*^2^) is used only for calculating *R*-factors(gt) *etc*. and is not relevant to the choice of reflections for refinement. *R*-factors based on *F*^2^ are statistically about twice as large as those based on *F*, and *R*- factors based on ALL data will be even larger.

### Fractional atomic coordinates and isotropic or equivalent isotropic displacement parameters (Å^2^)

**Table:**

	*x*	*y*	*z*	*U*_iso_*/*U*_eq_	Occ. (<1)
Cu1	0.55622 (4)	0.54229 (2)	0.70365 (2)	0.04278 (13)	
Cl2	0.3635 (8)	0.6509 (5)	0.6823 (4)	0.0600 (16)	0.8
Br2	0.3543 (11)	0.6580 (8)	0.6803 (5)	0.0397 (14)	0.2
Cl3	0.7542 (4)	0.6516 (3)	0.7331 (2)	0.0967 (10)	0.8
Br3	0.7538 (3)	0.6533 (2)	0.73299 (19)	0.0289 (6)	0.2
Cl4	0.4607 (10)	0.4267 (9)	0.7907 (6)	0.0538 (15)	0.8
Br4	0.4680 (16)	0.4110 (15)	0.7913 (9)	0.0511 (18)	0.2
Cl5	0.65337 (8)	0.43724 (5)	0.60629 (6)	0.0550 (2)	
N1	0.4441 (3)	0.2452 (2)	0.59512 (19)	0.0492 (6)	
H1	0.485 (3)	0.291 (2)	0.621 (2)	0.044 (9)*	
C2	0.4632 (3)	0.2199 (2)	0.5066 (2)	0.0457 (7)	
H2	0.5221	0.26	0.4752	0.055*	
C3	0.3953 (3)	0.1343 (2)	0.4620 (2)	0.0451 (7)	
C4	0.3078 (3)	0.0789 (2)	0.5126 (2)	0.0526 (8)	
H4	0.2593	0.0211	0.4842	0.063*	
C5	0.2909 (3)	0.1070 (2)	0.6036 (2)	0.0565 (8)	
H5	0.2323	0.0686	0.6367	0.068*	
C6	0.3615 (3)	0.1921 (2)	0.6445 (2)	0.0516 (7)	
H6	0.3519	0.2128	0.7061	0.062*	
C7	0.4157 (4)	0.1058 (2)	0.3622 (2)	0.0674 (9)	
H7A	0.5116	0.1301	0.3534	0.101*	
H7B	0.3362	0.1354	0.3139	0.101*	
H7C	0.4122	0.0338	0.3556	0.101*	
N8	0.8582 (3)	0.61568 (19)	0.52884 (19)	0.0494 (6)	
H8	0.820 (4)	0.599 (3)	0.574 (2)	0.082 (12)*	
C9	0.9467 (3)	0.6976 (2)	0.54021 (19)	0.0461 (7)	
H9	0.9599	0.735	0.5974	0.055*	
C10	1.0178 (3)	0.7265 (2)	0.46804 (19)	0.0446 (7)	
C11	0.9936 (3)	0.6688 (2)	0.3849 (2)	0.0522 (7)	
H11	1.0407	0.6864	0.3347	0.063*	
C12	0.9011 (4)	0.5857 (2)	0.3745 (2)	0.0587 (8)	
H12	0.885	0.5478	0.3176	0.07*	
C13	0.8329 (3)	0.5591 (2)	0.4483 (2)	0.0559 (8)	
H13	0.7703	0.5028	0.4427	0.067*	
C14	1.1163 (4)	0.8186 (2)	0.4800 (2)	0.0691 (9)	
H14A	1.0553	0.877	0.4589	0.104*	
H14B	1.1911	0.8115	0.4414	0.104*	
H14C	1.1654	0.8262	0.5472	0.104*	

### Atomic displacement parameters (Å^2^)

**Table:**

	*U*^11^	*U*^22^	*U*^33^	*U*^12^	*U*^13^	*U*^23^
Cu1	0.0438 (2)	0.0394 (2)	0.0472 (2)	−0.00244 (15)	0.01439 (16)	−0.00025 (15)
Cl2	0.060 (2)	0.051 (2)	0.073 (3)	0.0085 (14)	0.0232 (16)	−0.0012 (14)
Br2	0.046 (2)	0.040 (2)	0.036 (3)	0.0049 (18)	0.0167 (17)	0.0047 (16)
Cl3	0.097 (2)	0.091 (2)	0.104 (2)	−0.0196 (18)	0.0272 (18)	−0.0123 (19)
Br3	0.0263 (13)	0.0305 (15)	0.0315 (14)	−0.0156 (11)	0.0099 (11)	−0.0118 (12)
Cl4	0.0612 (13)	0.041 (3)	0.0660 (18)	0.0059 (13)	0.0280 (14)	0.0153 (12)
Br4	0.066 (3)	0.036 (4)	0.052 (2)	0.000 (2)	0.014 (2)	0.0125 (17)
Cl5	0.0605 (5)	0.0437 (4)	0.0693 (5)	−0.0081 (3)	0.0328 (4)	−0.0127 (4)
N1	0.0478 (15)	0.0433 (15)	0.0527 (16)	−0.0041 (13)	0.0029 (12)	−0.0027 (13)
C2	0.0449 (16)	0.0437 (16)	0.0494 (17)	−0.0037 (13)	0.0120 (13)	0.0060 (14)
C3	0.0444 (16)	0.0417 (16)	0.0473 (16)	0.0046 (13)	0.0054 (13)	0.0060 (13)
C4	0.0505 (18)	0.0368 (15)	0.066 (2)	−0.0078 (13)	0.0032 (15)	0.0033 (14)
C5	0.0518 (19)	0.0563 (19)	0.064 (2)	−0.0032 (15)	0.0180 (16)	0.0121 (16)
C6	0.0502 (18)	0.062 (2)	0.0449 (17)	0.0057 (15)	0.0141 (14)	0.0085 (15)
C7	0.087 (3)	0.060 (2)	0.054 (2)	0.0054 (18)	0.0111 (18)	−0.0029 (16)
N8	0.0457 (15)	0.0545 (16)	0.0509 (16)	0.0018 (12)	0.0167 (12)	0.0088 (13)
C9	0.0473 (17)	0.0496 (17)	0.0421 (16)	0.0010 (14)	0.0110 (13)	−0.0021 (13)
C10	0.0412 (15)	0.0520 (17)	0.0415 (16)	0.0037 (13)	0.0110 (12)	0.0065 (13)
C11	0.0509 (18)	0.064 (2)	0.0436 (17)	0.0096 (16)	0.0136 (14)	0.0052 (15)
C12	0.067 (2)	0.060 (2)	0.0457 (17)	0.0075 (17)	0.0055 (16)	−0.0093 (15)
C13	0.0502 (18)	0.0478 (18)	0.065 (2)	−0.0019 (14)	0.0025 (16)	−0.0008 (16)
C14	0.069 (2)	0.070 (2)	0.072 (2)	−0.0139 (18)	0.0230 (18)	0.0057 (18)

### Geometric parameters (Å, °)

**Table:**

Cu1—Cl2	2.232 (8)	C7—H7A	0.96
Cu1—Cl4	2.248 (10)	C7—H7B	0.96
Cu1—Cl5	2.2604 (8)	C7—H7C	0.96
Cu1—Cl3	2.273 (3)	N8—C13	1.336 (4)
Cu1—Br3	2.286 (2)	N8—C9	1.339 (4)
Cu1—Br2	2.356 (12)	N8—H8	0.82 (3)
Cu1—Br4	2.369 (17)	C9—C10	1.369 (3)
N1—C6	1.328 (4)	C9—H9	0.93
N1—C2	1.336 (4)	C10—C11	1.374 (4)
N1—H1	0.76 (3)	C10—C14	1.500 (4)
C2—C3	1.376 (4)	C11—C12	1.373 (4)
C2—H2	0.93	C11—H11	0.93
C3—C4	1.385 (4)	C12—C13	1.363 (4)
C3—C7	1.502 (4)	C12—H12	0.93
C4—C5	1.373 (4)	C13—H13	0.93
C4—H4	0.93	C14—H14A	0.96
C5—C6	1.362 (4)	C14—H14B	0.96
C5—H5	0.93	C14—H14C	0.96
C6—H6	0.93		
			
Cl2—Cu1—Cl4	97.5 (3)	C4—C5—H5	120.6
Cl2—Cu1—Cl5	135.34 (14)	N1—C6—C5	119.0 (3)
Cl4—Cu1—Cl5	99.0 (3)	N1—C6—H6	120.5
Cl2—Cu1—Cl3	100.2 (2)	C5—C6—H6	120.5
Cl4—Cu1—Cl3	135.8 (2)	C3—C7—H7A	109.5
Cl5—Cu1—Cl3	96.18 (8)	C3—C7—H7B	109.5
Cl2—Cu1—Br3	99.70 (18)	H7A—C7—H7B	109.5
Cl4—Cu1—Br3	136.0 (2)	C3—C7—H7C	109.5
Cl5—Cu1—Br3	96.53 (7)	H7A—C7—H7C	109.5
Cl3—Cu1—Br3	0.49 (16)	H7B—C7—H7C	109.5
Cl2—Cu1—Br2	0.6 (3)	C13—N8—C9	123.1 (3)
Cl4—Cu1—Br2	98.1 (3)	C13—N8—H8	119 (2)
Cl5—Cu1—Br2	135.17 (16)	C9—N8—H8	117 (2)
Cl3—Cu1—Br2	99.7 (2)	N8—C9—C10	120.3 (3)
Br3—Cu1—Br2	99.2 (2)	N8—C9—H9	119.9
Cl2—Cu1—Br4	101.6 (5)	C10—C9—H9	119.9
Cl4—Cu1—Br4	4.5 (7)	C9—C10—C11	117.4 (3)
Cl5—Cu1—Br4	94.7 (4)	C9—C10—C14	120.6 (3)
Cl3—Cu1—Br4	135.7 (3)	C11—C10—C14	122.0 (3)
Br3—Cu1—Br4	135.9 (3)	C12—C11—C10	121.2 (3)
Br2—Cu1—Br4	102.2 (5)	C12—C11—H11	119.4
C6—N1—C2	123.7 (3)	C10—C11—H11	119.4
C6—N1—H1	116 (2)	C13—C12—C11	119.6 (3)
C2—N1—H1	120 (2)	C13—C12—H12	120.2
N1—C2—C3	119.8 (3)	C11—C12—H12	120.2
N1—C2—H2	120.1	N8—C13—C12	118.4 (3)
C3—C2—H2	120.1	N8—C13—H13	120.8
C2—C3—C4	116.9 (3)	C12—C13—H13	120.8
C2—C3—C7	120.0 (3)	C10—C14—H14A	109.5
C4—C3—C7	123.1 (3)	C10—C14—H14B	109.5
C5—C4—C3	121.7 (3)	H14A—C14—H14B	109.5
C5—C4—H4	119.1	C10—C14—H14C	109.5
C3—C4—H4	119.1	H14A—C14—H14C	109.5
C6—C5—C4	118.8 (3)	H14B—C14—H14C	109.5
C6—C5—H5	120.6		

### Hydrogen-bond geometry (Å, °)

**Table:**

*D*—H···*A*	*D*—H	H···*A*	*D*···*A*	*D*—H···*A*
N1—H1···Cl5	0.76 (3)	2.50 (3)	3.158 (3)	145 (3)
N8—H8···Cl3	0.82 (3)	2.53 (3)	3.245 (4)	147 (3)
N8—H8···Cl5	0.82 (3)	2.72 (3)	3.332 (3)	133 (3)
